# Investigating the requirement for nasogastric tube feeding following transoral robotic surgery for head and neck cancer in Oxford: a retrospective cohort study

**DOI:** 10.1017/S0022215125000076

**Published:** 2025-06

**Authors:** Oliver Jones, Priyamal Silva, Stuart Winter

**Affiliations:** 1 School of Medicine and Biomedical Sciences, Medical Sciences Division, University of Oxford, Oxford, UK; 2 Blenheim Head and Neck Unit, NHS Foundation Trust, Oxford University, Oxford, UK; 3 Nuffield Department of Surgical Sciences, Medical Sciences Division, University of Oxford, Oxford, UK

**Keywords:** carcinoma head, neck, oropharynx, robotics

## Abstract

**Objectives:**

Transoral robotic surgery is a minimally invasive technique used in the management of head and neck cancer, though post-operative odynophagia can be a significant issue. There is debate about the necessity of elective nasogastric tube placement during the peri-operative period. This study examines the proportion of patients requiring elective nasogastric tube placement and evaluates whether pre-operative factors predict the need for nasogastric tube feeding.

**Methods:**

Data from patients who underwent transoral robotic surgery in Oxford were analysed to assess correlations between pre-operative factors and nasogastric tube feeding.

**Results:**

Fifty-three patients undergoing transoral robotic surgery underwent elective nasogastric tube placement; 43 per cent required the nasogastric tube for feeding or medication. Multivariate analysis showed significant associations between nasogastric tube feeding and sex (*p* = 0.028), peri-neural invasion (*p* = 0.024), tumour size (*p* = 0.012) and concurrent neck dissection (*p* = 0.019).

**Conclusion:**

Although nearly half of the patients benefited from elective nasogastric tube placement, the remainder did not. Benefits and risks of elective nasogastric tube placement should be carefully considered.

## Introduction

Due to significant morbidity and mortality associated with open head and neck surgery, there has been a shift, over recent decades, towards minimally invasive surgical techniques in head and neck cancer.^1^^,^^2^ Transoral robotic surgery (TORS) has been well-established in head and neck cancer management. Currently in the UK, TORS is performed for multiple surgical procedures including, but not limited to, radical tonsillectomy, tongue base tumour resection, tongue base mucosectomy and supraglottic laryngectomy.^3^ Transoral resections offer potential advantages to more conventional open surgery. Such advantages include improved post-operative cosmesis,^4^ shorter operative duration^5^ and a reduced hospital stay.^6^ Despite this, there remains an ongoing debate regarding the short- and long-term functional outcomes following TORS.^7^ Whilst ongoing studies are exploring the long-term impact on swallowing after TORS, the short-term effects, particularly those related to pain, have received less attention. Nasogastric tubes (NGTs) can be placed to ensure adequate nutritional intake and enteral drug delivery which may be compromised due to odynophagia. However, there is no clear guidance regarding the elective peri-operative placement of an NGT. Potential advantages of elective placement include earlier enteral feeding; however, this must be balanced with the potential economic and clinical disadvantages of prophylactic NGT placement. This study is a retrospective single-centre review of the use of elective placement of an NGT intra-operatively. The study aims to determine the proportion of patients who had an intra-operative NGT placed during TORS and later required post-operative feeding. Additionally, it seeks to identify whether specific patient, disease, or operative factors can predict the need for post-TORS NGT feeding (NGTF), enabling targeted NGT placement in patients at higher risk.

## Materials and methods

This study was approved by the Audit Department of Oxford University Hospitals NHS Foundation Trust on 21/03/2024 (Audit number 9285).

### Cohort selection

All patients undergoing TORS at the Blenheim Head and Neck Unit, Oxford, between April 2021 and June 2023 were identified. Patients were included in the study if they were over the age of 18 years and had TORS for head and neck cancer. Patients who had a day case procedure such as a biopsy, or resection of a benign lesion were excluded from the study. Of the patients who had an intra-operative NGT placed, post-operative NGTF was assessed in association with patient age, sex, smoking and alcohol status and number of co-morbidities. In addition, post-operative NGTG was assessed in association with procedure type, if there was a concurrent neck dissection performed, operative duration, T/N stage, primary size (mm), human papilloma virus (HPV) status, lymphovascular/peri-neural invasion, extracapsular spread, other treatment, complications and length of stay.

Data were collected retrospectively from patient medical records and stored anonymously on a spreadsheet in Microsoft Excel v. 16.87.

### Statistical analysis

Each continuous variable was first assessed for normality using a Shapiro–Wilk test. Univariate analyses were performed for each variable. For continuous data, an independent *t*-test was used, and for categorical data, either a chi-squared test of independence (if n > 5) or a Fisher’s exact test (if n ≤ 5) was used.

Due to the sparsity of data, for multivariate analysis, Firth’s bias-reduced logistic regression was used to determine if combinations of variables could predict NGTF. Replicating a previous study by Plonowska *et al*.^8^ where possible, variables included were patient age, sex, whether a neck dissection was also performed, intra-operative complications, primary site, primary size, T/N stage, HPV status, peri-neural invasion and extra-nodal extension. Univariate analysis was performed using IBM SPSS Ver. 29.0.2.0, and multivariate Firth’s bias-reduced logistic regression was performed using R Ver. 4.4.1. Statistical significance was defined as *p* less than 0.05.

## Results and analysis

### Patient demographics

Sixty-six patients were initially identified as being listed for TORS in Oxford University Hospitals Trust. However, six of these did not undergo TORS after an intra-operative assessment revealed more extensive disease. Seven patients were excluded as they had minor day-case surgery. This resulted in 53 patients being included in the study. Forty (75 per cent) were male and 13 (25 per cent) were female. The mean age of patients was 60 (range = 28–84). All 53 patients had TORS using a Da Vinci surgical X robotic platform. Patient demographics are shown in Table 1.

**Table 1.** Overall demographics of patients included in this study

Demographic	NGT not used	NGT used	Significance
Sex			
Male	24	16	n/s*
Female	6	7	
Age (years)			
Mean	59	61	n/s**
Range	28−84	47−76	
Tobacco smoking			
Never smoker	13	11	n/s***
Current smoker	5	3	
Ex-smoker	12	9	
Alcohol			
None	11	8	n/s***
1−5 units per week	7	6	
10−20 units per week	9	7	
>20 units per week	3	2	
Number of co-morbidities			
Median	1	0	n/s***
Range	0−6	0−4	

*Note*: Patients have been sub-divided into whether an NGT was used post-TORS or not.

* Chi-squared test of independence.

** Independent t-test.

*** Fisher’s exact test.

Abbreviations: n/s = not significant; NGT = nasogastric tube; TORS = transoral robotic surgery.

### Post-TORS NGTF

Of the 53 patients included in this study, all of whom had an NGT placed intra-operatively, 57 per cent did not use their NGT and regained swallowing function post-operatively. Of the remaining 23 patients who did require post-operative NGTF, 23 per cent (6) used their NGT only for medication delivery and regained oral feeding after the immediate post-operative recovery period. The remaining 17 patients required post-operative enteral feeding. The mean length of stay for the whole cohort was 5 days (range = 1–15 days). Of those that used their NGT, the mean length of stay was 7 days (range = 1–15 days), and of those who did not use their NGT, the mean length of stay was 4 days (range = 1–9 days). The increase in length of stay for those who did require NGTF compared to those who did not was statistically significant (independent *t*-test, t(29) = −2.373; *p* = 0.024). Of the patients who did require NGTF, the primary cause was odynophagia; however, no patients were readmitted due to pain following discharge. The mean duration that the NGT was in situ was 7 days (SD ± 5.45). This was a significantly longer duration than in those who did not use their NGT (mean = 1.8 days; independent *t*-test, t(23) = 4.54; *p* < 0.001). No patient required long-term NGTF.

### Predicting post-operative NGTF using pre-operative factors

In our univariate analysis, clinico-pathological characteristics were independently assessed to determine their association with NGTF after TORS. Patient characteristics such as sex, age, smoking, alcohol and co-morbidities were not significantly associated with NGTF (Table 1). The size of the primary tumour was correlated with post-TORS NGTF; however, this was not a significant association. Additionally, no significant correlation was identified between any tumour or treatment characteristics and post-TORS NGTF (Tables 2 and 3).

**Table 2.** Patient tumour characteristics

Characteristic	NGT not used	NGT used	Significance
Primary site			
BOT/lingual tonsil	9	7	n/s***
Posterior pharyngeal wall	1	1	
Tonsil	8	8	
Unknown	12	7	
Primary size (mm)			
Mean	15.8	21.8	n/s**
SD	11.2	7.5	
Tumour stage			
T0	12	8	n/s***
T1	13	8	
T2	4	6	
T3	1	1	
Nodal stage			
N0	2	2	n/s***
N1	16	16	
N2	8	3	
N3	1	0	
Nx	3	2	
HPV status			
Positive	26	19	n/s***
Negative	4	4	
Peri-neural invasion			
Yes	2	3	n/s***
No	21	15	
Unknown	7	5	
Lymphovascular invasion			
Yes	6	2	n/s***
No	17	16	
Unknown	7	5	
Extra-nodal extension			
Yes	20	13	n/s***
No	3	4	
Unknown	7	6	

*Note*: Patients have been sub-divided into whether an NGT was used post-TORS or not.

*Chi-squared test of independence.

** Independent t-test.

*** Fisher’s exact test.

Abbreviations: BOT = base of tongue; HPV = Human papilloma virus; n/s = not significant; NGT = nasogastric tube; TORS = transoral robotic surgery.

**Table 3.** Procedure characteristics of patients included in this study

Characteristic	NGT not used	NGT used	Significance
Procedure			
BOT resection (BL)	14	7	n/s***
BOT resection (UL)	8	9	
Oropharyngectomy	10	8	
Posterior pharyngeal wall	1	1	
Concurrent neck dissection			
Bilateral	2	1	n/s***
Ipsilateral	19	16	
No	9	6	
Procedure time (minutes)			
Mean	225	266	n/s**
SD	104.3	123.2	

*Note*: Patients have been sub-divided into whether an NGT was used post-TORS or not.

*Chi-squared test of independence.

** Independent t-test.

*** Fisher’s exact test.

Abbreviations: BL = bilateral; BOT = base of tongue; n/s = not significant; NGT = nasogastric tube; SD = standard deviation; TORS = transoral robotic surgery; UL = nilateral.

For multivariate analysis, it was planned to include any variables that were statistically significantly associated with post-TORS NGTF on univariate analysis. However, as no statistically significant findings were identified, where possible, variables included in the multivariate logistic regression performed by Plonowska *et al.* were used.^8^ Here, included variables were age, sex, neck dissection, complications, primary site, tumour stage, nodal stage, HPV status, peri-neural invasion, extra-nodal extension and primary size. Due to a significant risk of sparse data bias, Firth’s bias-reduced logistic regression was utilised (Table 4). Here, sex (95 per cent confidence interval [CI]: 0.234–5.503; *p* = 0.028), peri-neural invasion (95 per cent CI:0.498–8.697; *p* = 0.024), primary tumour size (95 per cent CI: 0.023–0.219; *p* = 0.012) and whether the patient had a concurrent neck dissection (95 per cent CI: −7.636 – −0.516; *p* = 0.019) were significantly associated with post-operative NGTF.

**Table 4.** Firth’s bias-reduced logistic regression analysis

Characteristic	Regression coefficient	SE (coefficient)	Chi-squared	95% CI (lower)	95% CI (upper)	*P*-value
Age (years)	−0.118	0.070	2.811	−0.294	0.019	0.094
Sex	2.412	1.158	4.807	0.234	5.503	**0.028**
Neck dissection	−3.662	1.634	5.471	−7.636	−0.516	**0.019**
Complications	0.933	1.065	0.688	−1.347	3.258	0.407
Primary site	0.632	0.539	1.344	−0.431	1.911	0.246
Tumour stage	−1.421	0.763	3.542	−3.161	0.057	0.059
Nodal stage	−0.823	0.436	3.730	−1.880	0.011	0.053
HPV status	0.440	1.259	0.107	−2.676	3.085	0.744
Peri-neural invasion	4.056	1.879	5.126	0.498	8.697	**0.024**
Extra-nodal extension	−1.001	0.888	1.181	−2.936	0.821	0.277
Primary size (mm)	0.109	0.045	6.367	0.023	0.219	**0.012**

Abbreviations: CI = confidence interval; HPV = Human papilloma virus; SE = standard error.

## Discussion

TORS has significantly improved short- and long-term functional outcomes compared to conventional open surgery when treating head and neck cancer. In a study by Iseli *et al.* of 54 patients undergoing TORS for resection of head and neck cancer,^7^ only three patients stayed in hospital for longer than 1 week (due to aspiration pneumonia in two and salivary fistula in one). A tracheostomy was used in five patients. Additionally, long-term swallowing outcomes were also acceptable with a mean pre-operative MD Anderson Dysphagia Inventory (MDADI) score of 75/100 compared with a mean post-operative MDADI score of 65/100.^7^ Early post-operative swallowing may also be associated with improved long-term swallowing outcomes amongst those with oral cavity cancer.^9^ Mettias *et al.* showed in a study of 104 patients who had TORS that more than 75 per cent of patients maintained normal liquid intake immediately after surgery, and NGTF was required in just five patients. Just one of these required long-term NGTF. After one year, normal liquid and dietary intake was achieved in 98 per cent of patients.^10^ It should be noted that the cohort in this study were all oropharyngeal resections rather than oral cavity tumours. TORS is now routine in many centres globally; however, there remains no censuses on the need for a post-operative NGT. Whilst NGTs serve as an easy route of delivery for nutrition and medications in the dysphagic patient, they may also serve as a source of discomfort, are resource intensive and environmentally impactful. Additionally, local protocols require that NGT positioning is confirmed on X-ray by a consultant radiologist. Due to the lack of consensus guidelines, management options include the elective placement of an NGT for all patients, or selecting patients based on the perceived post-operative need. This could be influenced by pre-morbid swallowing function, the extent of surgery or combined factors such as if the patient has a concurrent neck dissection. Placing an NGT intra-operatively has the advantages of maximising the likelihood of its safe positioning and avoiding the need of passing it alongside a new surgical site in the post-operative period. This would prevent any unnecessary trauma caused by post-operative placement. Alternatively, an NGT could be placed post-operatively as a reactive measure for those struggling to swallow. Reactive NGT placement ensures that only patients who require NGTF receive an NGT. This reduces unnecessary discomfort, pain and radiation exposure from a chest X-ray for those who do not require one. Additionally, reactive NGT placement may reduce the overall cost and recourses of NGT management. The risks and benefits must be carefully considered before deciding the appropriate management of NGT placement for patients undergoing TORS. Due to the lack of consensus data, there is no defined protocol as for whom an NGT is beneficial. A study by Plonowska *et al*. of 95 patients from a single centre, 49.5 per cent had an NGT placed intra-operatively. Of these, 36 per cent required NGTF. Of the 50.5 per cent who did not have an NGT placed intra-operatively, 11.6 per cent required NGTF.^8^ Plonowska *et al*. also identified tumour size and bilateral neck dissections as being predictive for NGTF. Data from this study suggest that primary tumour size, neck dissection, peri-neural invasion and sex were significant predictors of post-operative NGTF. Despite this, in this study, data were sparse, and a larger cohort would be required to more accurately predict post-operative NGTF need. Additionally, post-operative odynophagia was the primary cause of swallowing difficulties. Therefore, it is possible that, by optimising post-TORS analgesia, the proportion of patients requiring NGTF would be minimised, reducing the need to predict NGTF based on pre-operative factors. This study has several limitations. The cohort size was small, and the patients were all treated within Oxford University Hospitals Foundation Trust. However, in line with other published work there is an emerging set of data to suggest that certain factors might predict the need for post-operative NGTF. There is a need for future prospective cohort studies to determine the use and safety profile of reactive NGT placement following TORS.

- There is ongoing debate regarding the short- and long-term functional outcomes following transoral robotic surgery
- There remains no consensus guidance on the role for nasogastric tube (NGT) feeding in the immediate post-operative period after transoral robotic surgery for head and neck cancer
- Almost half of patients do benefit from elective NGT placement, but the remainder do not require it
- Benefits and risks of elective NGT placement should be carefully considered
- Additional studies with larger cohorts are required to determine the role for peri-operative NGT placement in transoral robotic surgery
